# The impact of ischemic reperfusion injury on contralateral kidneys and the determinants of renal prognosis after robot-assisted partial nephrectomy

**DOI:** 10.1371/journal.pone.0321769

**Published:** 2025-04-15

**Authors:** Mitsunori Matsuo, Kensei Taguchi, Yunosuke Yokota, Kei Fukami, Tsukasa Igawa

**Affiliations:** 1 Department of Urology, Kurume University School of Medicine, Kurume, Fukuoka, Japan; 2 Division of Nephrology, Department of Medicine, Kurume University School of Medicine, Kurume, Fukuoka, Japan; Khalifa University of Science and Technology, UNITED ARAB EMIRATES

## Abstract

Robot-assisted laparoscopic partial nephrectomy (RAPN) is a safe and effective option for renal cell carcinoma (RCC). However, clamping of renal artery during RAPN sometimes causes ischemic reperfusion (IR) injury (IRI), which affects renal function at some later time. In the present study, we inserted catheters into the bilateral ureters from before RAPN until 24 hours after and analyzed urine biomarkers of renal injury excreted from both resected and contralateral kidneys to determine and investigated which biomarkers predict the future decline in renal function in patients with RCC and rodent IR model. Twenty-three patients diagnosed with RCC (66.4 ± 10.8 years old, eGFR: 73.6 ± 15.3 mL/min/1.73m^2^) were enrolled and ureteral catheters were inserted in both ureters. Urinary neutrophil gelatinase-associated lipocalin (NGAL), beta-2-microglobulin (β₂MG), N-acetyl-β-D-glucosaminidase were measured at several time points. Gene expression of injury markers in contralateral kidneys were analyzed in unilateral IR rodents. All the urinary markers were elevated 30 minutes after the clamping and sustained high until 24 hours in resected kidneys. Meanwhile, urinary NGAL and β_2_MG excreted from contralateral kidneys increased at 6 and 24 hours after the clamping. Warm ischemic time, estimated blood loss, and excised kidney weight were not associated with renal dysfunction; however, only contralateral urinary β_2_MG at 6 hours was correlated. *Ngal* and *Il-6* mRNA in contralateral kidneys were upregulated in unilateral IR rodents. RAPN-related IRI induces contralateral kidney injury. Contralateral urinary β_2_MG can become a potent biomarker to predict the onset of kidney injury after RAPN.

## Introduction

The incidence of renal cell carcinoma (RCC) has increased in recent years. Although nephrectomy has been considered one of the standard surgical treatment for RCC regardless of tumor size, EORTC-30904 trial recently reported that partial nephrectomy for small-diameter RCC showed no significant difference in overall survival compared to radical nephrectomy [1,2]. Further, partial nephrectomy was shown to reduce the future incidence of chronic kidney disease (CKD) and cardiovascular events reported by the National Comprehensive Cancer Network, the European Association of Urology, and the American Association of Urology guidelines. Thus, partial nephrectomy has drawn the attention as the first-line surgical option for small-diameter RCC [3–6]. Also, robot-assisted laparoscopic partial nephrectomy (RAPN) can save the time to perform hemoperfusion when compared to laparoscopic partial nephrectomy (LPN) [3–7], suggesting that RAPN can be considered a safe and effective therapeutic option for patients with RCC. However, RAPN sometimes causes transient kidney injury due to ischemia-reperfusion (IR) in resected kidneys because RAPN requires warm occlusion by renal artery clamping during removing RCC. Therefore, evaluation of biomarkers and mechanisms regarding IR-associated kidney injury may be necessary to improve the postoperative renal outcome.

Urinary injury biomarkers such as β_2_-microglobin (β_2_MG), N-acetyl-β-D-glucosaminidase (NAG), and neutrophil gelatinase-associated lipocalin (NGAL) has been widely used for the early diagnosis of acute kidney injury (AKI) [8]. Indeed, Akpinal et al. have reported that ischemic AKI after nephron-sparing surgery can be detected early by urinary NGAL measurement [8]. However, postoperative renal prognosis and predictive markers by the IR injury (IRI) during RAPN and its effect on the contralateral kidney remain unknown. In the present study, we inserted ureteral catheters in both resected and contralateral ureter and collected urine at several time points to determine the effect of IR by the renal artery clamping on renal dysfunction, and investigated which urinary biomarkers can predict postoperative decline in kidney function in patients receiving RAPN. To confirm whether contralateral kidneys would be impaired when another kidney undergoes IRI, the gene expression of renal injury markers in the contralateral kidneys were analyzed in rodent unilateral IR model.

## Materials and methods

### Operation methods

Twenty-three patients with clinically diagnosed RCC between 1^st^ August 2019 and 31^th^ March 2020 were prospectively enrolled in this study. All patients were placed ureteral catheters in the ureter on the resected and contralateral sides before the start of RAPN and confirmed urethral patency during tumor resection. In standard RAPN procedures, a ureteral catheter is inserted transurethrally in all cases. In this study, after providing patients with explanations regarding potential side effects, such as bleeding, ureteral catheters were inserted into the resected and contralateral kidneys. All catheters on both sides were removed 24 hours after IR. No significant disadvantages or adverse effects from this procedure were observed in the patients. Tumor resection was performed with total renal artery clamping, and after tumor dissection, an inner suture was performed on the resected surface including the open ureter, and renal parenchymal suture (renorrhaphy) was performed after removal of the occlusion. All ureteral catheters were removed after the final specimen collection on the following day.

Urine was collected from bilateral catheters before occlusion and at 30, 60 minutes, 6 and 24 hours after IR. Serum was also collected at the same time. Urinary NGAL (chemiluminescent immunoassay), NAG (colorimetric method), and β_2_MG (latex agglutination immunoassay) were measured to evaluate renal impairment, and all were corrected by urinary creatinine (Cr). Serum blood urea nitrogen (BUN), Cr, and cystatin C (CysC) were also measured as markers of renal function. These parameters were analyzed by commercially available laboratories. Data on the clinical characteristics of the participants, including age, body weight, body mass index, mean systolic blood pressure, and R.E.N.A.L Nephrometry Score (RNS) were obtained from the electronic medical records of Kurume University Hospital. eGFR was calculated using a previously described [9]. The same hydration protocol procedure before and after the RAPN was performed. In all patients, written preoperative consent for ureteral catheterization and the use of data was obtained. The study was approved by the Ethics Committee of the Kurume University School of Medicine for human research (Ethical No. 19093) and animal research (2023–163) and was performed in accordance with the Declaration of Helsinki. The data for this study obtained from the patients’ medical records were anonymized so that the patients could not be identified. Human rights are well-protected.

### Animal experiments

10-week-old C57BL/6J mice (The Jackson Laboratory Japan, Yokohama, Japan) were anesthetized by the injection with Medetomidine hydrochloride (0.3 mg/kg; Cat No. SMB01393, Sigma-Aldrich), midazolam (4 mg/kg; Cat No. 10385, Fuji Film-Wako), and butorphanol tartrate (5 mg/kg, Cat No. B9156, Sigma-Aldrich). After cutting the ventral median line skin and muscle layer, the upper and lower poles of the kidney was dissected free from surrounding tissue. After liberating the kidney from surrounding tissue, the left kidney pedicle was clamped for 30 minutes using a clamp holder. After removal of the vascular clamp, the muscle layer was closed by using absorbable suture and then skin was closed by using monofilament nylon non-absorbable suture. For creating sham mice, we cut the ventral median line skin and muscle layer, and then close the skin and muscle layer in a same way as IRI mice. We closely monitored the mice of both groups and administrated with 150 μL of Buprenorphine (0.15mg/kg, Nissin Pharmaceutical Co., Ltd) every 12 hours until day 2 for pain and discomfort. For sacrifice, the mice were anesthetized after the exposure to isoflurane and both kidneys were removed 24 hours and seven days after the induction of IR.

### Real-time PCR

Mice were sacrificed on days 1 and 7 after the IR surgery. The collected right kidney (contralateral side) was homogenized after removing the capsule. Total RNA extracted from each kidney cortex was used to synthesize cDNA with iScript™ cDNA Synthesis Kit (Bio-Rad, 170891). Real-time PCR was performed with iTaq Universal SYBR Green (Bio-Rad, 1725121). Glyceraldehyde-3-phosphate dehydrogenase was used as an internal control. The relative mRNA expression of each gene was calculated using the ΔΔCt method. Primers for targeted genes were purchased from Applied Biosystems (MA, USA).

### Statistical analysis

To compare the urinary renal injury markers before and after the IR, one-way analysis of variance followed by post hoc multiple comparison analysis (Steel-Dwass test) was used to assess the differences among the groups. To determine the correlation with the changes in eGFR at a month, univariate regression analysis was performed. Human data are presented as mean ± standard deviation. Animal data are presented as mean ± standard error of the mean. A p-value < 0.05 was considered statistically significant. All statistical analyses were performed using JMP Pro ver. 16 Software (SAS Institute Inc.).

## Results and discussion

Clinical background of the enrolled patients is shown in Table 1. The mean age was 66.4 ± 10.8 years and the mean body mass index was 25.6 ± 3.5. The tumor was detected in right kidneys in 8 patients and in left kidneys in 15 patients. The mean tumor diameter was 26.4 ± 6.2 mm, and the mean R.E.N.A.L Nephrometry Score was 7.2 ± 1.1 points. Preoperative renal function was BUN: 15.5 ± 5.9 mg/dL, Cr: 0.78 ± 0.18 mg/dL, eGFR: 73.4 ± 15.2 mL/min/1.73m^2^.

**Table 1:** Clinical characteristics of the patients before RAPN.

Variables	Mean ± SD (IQR)
No. of patients	23
Age (years old)	66.4 ± 10.8 (43-82)
Sex (No.) (male/female)	16/7
Side with the cancer (No.) (right/left)	8/15
Body weight (kg)	65.9 ± 12.7 (42.8-100.6)
Body mass index (kg/m^2^)	25.6 ± 3.5 (19.0-37.0)
Tumor diameter (mm)	26.4 ± 6.2 (15-36)
RENAL Nephrometry Score (points)	7.2 ± 1.1 (5-9)
Mean systolic blood pressure (mmHg)	129.1 ± 12.5 (105-150)
Hemoglobin (g/dl)	14.2 ± 1.6 (10.3-16.5)
Serum albumin (g/dl)	4.32 ± 0.25 (4.0-4.8)
Blood urea nitrogen (mg/dl)	15.5 ± 5.9 (9.0-37.0)
Serum creatinine (mg/dl)	0.78 ± 0.18 (0.54-1.42)
eGFR (ml/min/1.73m^2^)	73.4 ± 15.2 (30.4-99.6)
Uric acid (mg/dl)	5.98 ± 1.12 (3.7-8.4)
Total cholesterol (mg/dl)	202 ± 40.0 (85-276)

Values are shown as mean ± SD and range. RAPN= robot-assisted laparoscopic partial nephrectomy, SD=standard deviation, IQR=interquartile range, No.=number, eGFR=estimated glomerular filtration rate

There were no differences in NGAL, β_2_MG, and NAG in urine collected from both resected and contralateral kidneys before renal artery clamping (Table 2).

**Table 2:** Results of urinary renal injury markers before RAPN.

Preoperative urine markers	Mean ± SD (IQR)	P value
Urinary NGAL (μg/ g Cr)		
Resected kidney	0.10 ± 0.24 (0-1.15)	
Contralateral kidney	0.07 ± 0.14 (0-0.61)	n.s.
Urinary β_2_MG (μg/ mg Cr)		
Resected kidney	1.23 ± 4.77 (0.74-231)	
Contralateral kidney	1.17 ± 4.22 (0.81-205)	n.s.
Urinary NAG (IU/ g Cr)		
Resected kidney	0.12 ± 0.10 (0.01-0.45)	
Contralateral kidney	0.12 ± 0.11 (0.01-0.43)	n.s.

Values are shown as mean ± SD and range. SD=standard deviation, IQR=interquartile range, NGAL=Neutrophil gelatinase-associated lipocalin, Cr=creatinine, β_2_MG=β_2_ microglobulin, NAG=N-acetyl-β-D-glucosaminidase

The results of surgical procedure were as follows: total operative time: 191 ± 37.8 minutes; console time: 101.3 ± 31.5 minutes; warm ischemic time: 15.4 ± 3.6 minutes; total blood loss: 21.6 ± 29.4 ml. Postoperative complications were postoperative hemorrhage and hydronephrosis in one patient each (4.3%), and no inter-allogeneic blood transfusion or laparotomy surgery was performed. Histopathological results showed clear cell RCC in 20 patients (87.0%), chromophobe RCC in 2 patients (8.7%), and angiomyolipoma in 1 patient (4.3%) with no positive margins (Table 3).

**Table 3:** Data of RAPN.

Results of RAPN		Mean ± SD (IQR)
Total operative time (min)		191.3 ± 37.8 (134-251)
Laparoscopic time (min)		141.7 ± 30.1 (102-213)
Console time (min)		101.3 ± 31.5 (53-181)
Warm ischemic time (min)		15.4 ± 3.6 (11-23)
Estimated bleeding loss (ml)		21.6 ± 29.4 (5-115)
Resected weight (g)		13.1 ± 10.1 (2-47)
Postoperative complications (cases)	Bleeding Hydronephrosis	1 (4.3%) 1 (transient) (4.3%)
Pathological results (cases)	ccRCC Chromophobe AML	20 (87.0%) 2 (8.7%) 1 (4.3%)

Values are shown as mean ± SD and range. SD=standard deviation, IQR=interquartile range, RAPN=Robot-assisted laparoscopic partial nephrectomy, ccRCC=clear cell renal cell carcinoma, AML=angiomyolipoma

**Fig 1:**
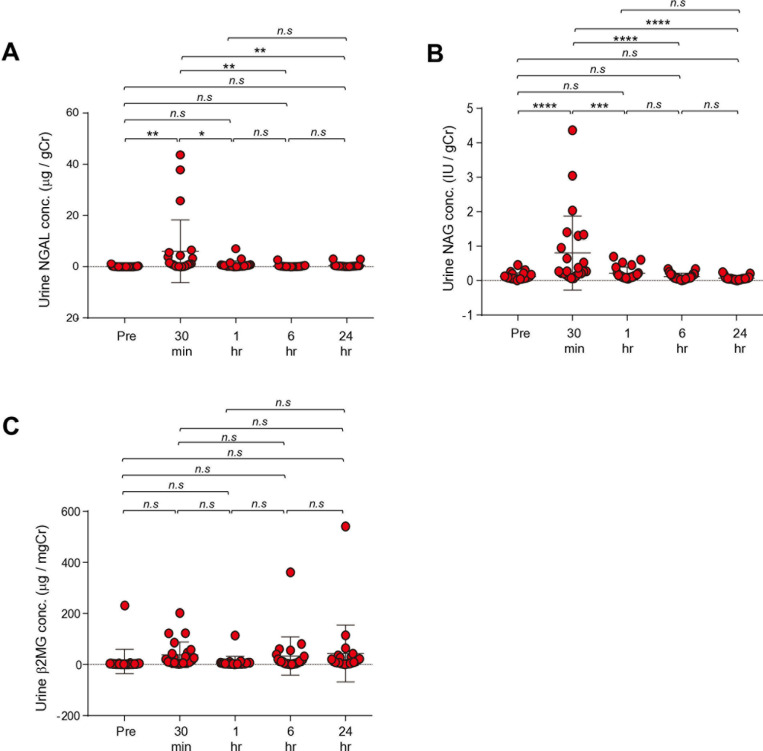
Urinary renal injury markers before and after IR by RAPN in the resected kidney. NGAL/Cr ratio **(a)**, β_2_MG/Cr ratio **(b)**, and NAG/Cr ratio **(C)**. ^***^ p<0.001, ^**^ p<0.01 vs pre, ^###^ p<0.001, ^#^ p<0.05 vs post 30 min. IR, ischemic reperfusion; RAPN, robot-assisted partial nephrectomy; NGAL, Neutrophil gelatinase-associated lipocalin; Cr, creatinine; β_2_MG, β_2_-microglobulin; NAG, N-acetyl-β-D-glucosaminidase.

All of the renal injury markers in urine from the resected kidneys were significantly elevated from 30 minutes to 24 hours after renal artery clamping. The increase in urine concentration of NGAL and β_2_MG continued until 24 hours (NGAL: 30 min; p=0.0001 vs pre, 60 min; p<0.001 vs pre, 6 hrs; p<0.01 vs pre, 24 hrs; p<0.001 vs pre, β_2_MG: 30 min; p<0.0001 vs pre, 60 min; p<0.05 vs pre, 6 hrs; p<0.01 vs pre, 24 hrs; p<0.0001 vs pre) (Table 4, Fig 1). NAG was elevated only 30 minutes after IR (NAG: 30 min; p=0.0003 vs pre, 60 min; p=0.129 vs pre, 6 hrs; p=0.999 vs pre, 24 hrs; p=0.165 vs pre) (Table 4, Fig 1). Urine NAG was lower at 24 hours than before IR (Table 4, Fig 1).

**Table 4:** Renal function and urinary injury markers corrected by creatinine before and after RAPN.

Serum markers of renal function					
	Pre	Post 30 minutes	Post 60 minutes	Post 6 hours	Post 24 hours
BUN (mg/ dL)	14.97 ± 5.69	15.44 ± 5.70	15.54 ± 5.90	13.81 ± 2.94	11.48 ± 4.76
Cr (mg/ dL)	0.820 ± 4.216	1.015 ± 0.244	1.020 ± 0.232	0.877 ± 0.174	0.836 ± 0.212
CysC (mg/ L)	0.917 ± 0.301	1.074 ± 0.336	1.014 ± 0.293	0.793 ± 0.131	0.809 ± 0.219
Resected kidney of urinary renal injury markers					
	Pre	Post 30 minutes	Post 60 minutes	Post 6 hours	Post 24 hours
NGAL (μg/ g Cr)	0.098 ± 0.242	6.036 ± 12.23	0.760 ± 1.499	0.271 ± 0.536	0.454 ± 0.791
β_2_MG (μg/ mg Cr)	1.227 ± 4.769	3.777 ± 5.035	0.910 ± 2.295	3.314 ± 7.454	4.306 ± 11.14
NAG (IU/ g Cr)	0.119 ± 0.102	0.800 ± 1.075	0.210 ± 0.039	0.094 ± 0.020	0.068 ± 0.060
Contralateral kidney of urinary renal injury markers					
	Pre	Post 30 minutes	Post 60 minutes	Post 6 hours	Post 24 hours
NGAL (μg/ g Cr)	0.069 ± 0.137	0.248 ± 0.536	0.101 ± 0.135	0.134 ± 0.185	0.150 ± 0.217
β_2_MG (μg/ mg Cr)	1.168 ± 4.216	0.891 ± 2.075	1.143 ± 2.935	7.100 ± 9.454	4.755 ± 9.129
NAG (IU/ g Cr)	0.124 ± 0.111	0.140 ± 0.167	0.183 ± 0.480	0.076 ± 0.077	0.051 ± 0.052

Values are shown as mean ± SD. SD=standard deviation, NGAL=Neutrophil gelatinase-associated lipocalin, β_2_MG=β_2_ microglobulin, NAG=N-acetyl-β-D-glucosaminidase, BUN=blood urea nitrogen, Cr=creatinine, CysC=cystatin C

**Fig 2:**
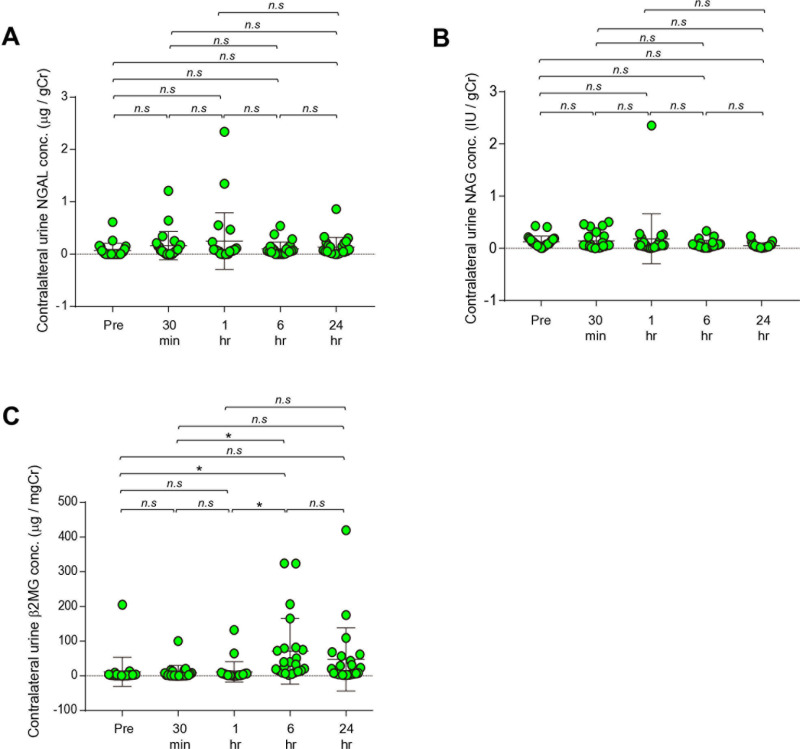
Urinary renal injury markers before and after IR by RAPN in the contralateral kidney. NGAL/Cr ratio **(a)**, β_2_MG/Cr ratio **(b)**, and NAG/Cr ratio **(C)**. ^***^ p<0.001, ^*^ p<0.05 vs pre, ^###^ p<0.001, ^##^ p<0.01 vs post 30 min. IR, ischemic reperfusion; RAPN, robot-assisted partial nephrectomy; NGAL, Neutrophil gelatinase-associated lipocalin; Cr, creatinine; β_2_MG, β_2_-microglobulin; NAG, N-acetyl-β-D-glucosaminidase.

With respect to the urine from the contralateral kidney, NGAL was elevated only 24 hours after renal artery clamping (NGAL: 30 min; p=0.333 vs pre, 60 min; p=0.412 vs pre, 6 hrs; p=0.075 vs pre, 24 hrs; p<0.05 vs pre). Further, urine β_2_MG was markedly elevated at 6 and 24 hours (β_2_MG: 30 min; p=0.998 vs pre, 60 min; p=0.373 vs pre, 6 hrs; p<0.0001 vs pre, 24 hrs; p<0.001 vs pre) (Table 4, Fig 2). The increase in NAG was not observed in the contralateral urine. Transient kidney injury assessed by serum Cr was found at 30 and 60 minutes after renal artery clamping (p<0.05 vs pre, respectively), whereas BUN and CysC were not. While serum BUN and Cr returned to the baseline at 6 hours; CysC was slightly lower at 6 and 24 hours than before IR (Table 4).

**Fig 3:**
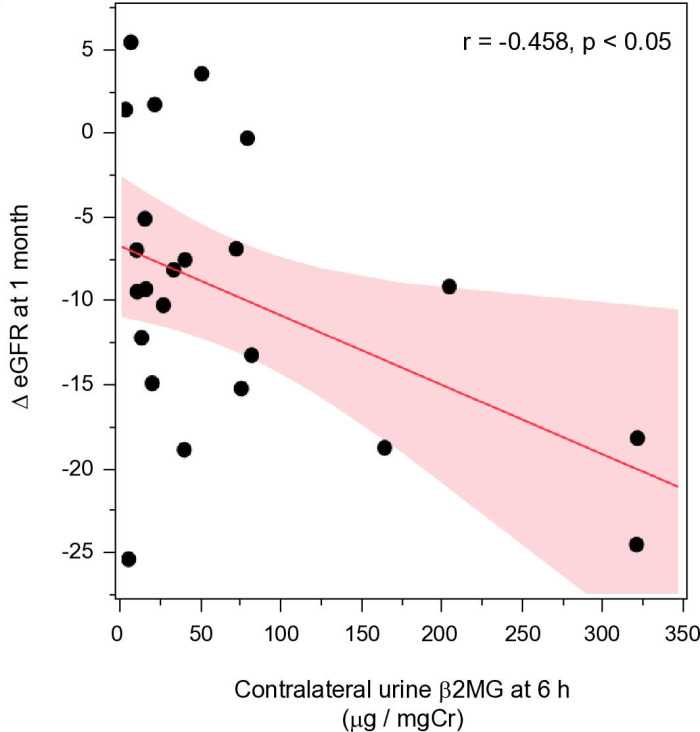
Univariate regression analysis for the correlation between eGFR changes at 1 month and Urinaryβ_2_MG/Cr ratio. β_2_MG, β_2_-microglobulin; Cr, creatinine; eGFR, estimated glomerular filtration ratio.

Among the urinary injury biomarkers, the contralateral urine β_2_MG at 6 hours after renal artery clamping is associated with the decline in renal function at 1 month (r=−0.458, p<0.05) (Fig 3). Meanwhile, none of the injury markers in urine excreted from the resected kidney, blood loss, and kidney excised weight were not associated with postoperative renal dysfunction.

**Fig 4:**
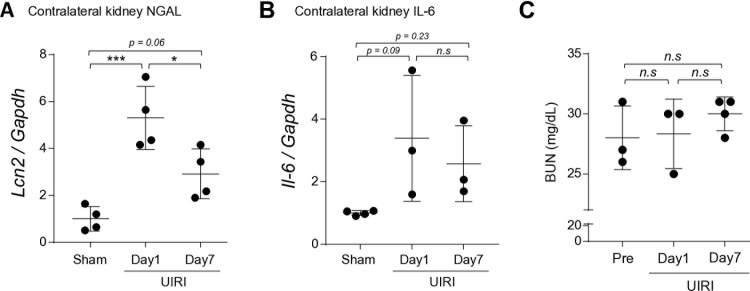
Contralateral renal NGAL (a) and IL-6 (b) gene expressions and BUN (c) levels in IRI C57BL/6j mice. ^***^ p<0.001 vs sham, ^*^ p<0.05 vs Day 7. NGAL, Neutrophil gelatinase-associated lipocalin; lcn2, lipocalin 2; IL-6, interleukin-6; BUN, blood urea nitrogen; Gapdh, glyceraldehyde-3-phosphate dehydrogenase; UIRI, unilateral ischemic reperfusion injury.

In unilateral IR rodents, we identified an increase in NGAL gene levels in the contralateral kidneys on day 1 when compared to the sham mice, which continued until day 7 (Fig 4a). IL-6, an inflammatory cytokine, showed a similar trend as NGAL mRNA expression (Fig 4b) in spite of no difference in renal function before and after the IRI (Fig 4c).

Interception of renal blood flow is a prerequisite process for RAPN; but, kidney injury is likely to occur immediately after interception of renal blood flow [10]. There have been no reports investigating whether contralateral kidney would be impaired when another kidney undergoes IR injury. Thus, we collected urine from resected and contralateral kidney separately during surgery of RAPN. We identified that renal artery clamping-induced IR injury cause contralateral kidney injury, which may be associated with the new onset of chronic injury.

NGAL, a 25 kDa protein secreted from secretory granules of human neutrophils, was known to be secreted from distal tubular epithelial cells in very early stages of AKI [11]. In an investigation of urinary biomarkers for kidney injury associated with RAPN, urinary NGAL concentration in the resected kidney can predict postoperative renal dysfunction in the patients receiving RAPN [12]. In our study, while urine NGAL was elevated in the resected kidney, tubular injury markers including NAG and β_2_MG were also increased in the urine from the contralateral kidney. Recently, Rooij et al. reported that kidney function significantly decreased and urinary β_2_MG levels increased 24 hours after unilateral nephrectomy in healthy kidney donors [13]. The unilateral nephrectomy-induced reduction in kidney mass increases blood pressure in the contralateral kidney, leading to elevated urinary β_2_MG levels. Considering that kidney function returned to baseline 24 hours postoperatively in our cases, the continuous increase in contralateral tubular injury markers is possibly attributable to IRI of the resected kidney rather than the reduction in kidney mass due to tumor removal. Akpinal et al. demonstrated that urinary NGAL levels increased significantly 3 hours after intraoperative total or renal artery clamping [8]. In contrast, in our cases, urinary NGAL and NAG levels were elevated 30 min after IR, but only from the resected kidney, indicating that IR-induced tubular injury due to renal artery clamping might occur immediately after IR rather than after 3 hours in the resected kidney. There was a time lag regarding the elevation of injury markers between resected kidney and contralateral kidney, suggesting that some humoral or neurogenic factors may extend renal damage to the contralateral kidney. Recently, multiple organ crosstalk has been reported. For instance, IR-induced AKI causes lung injury through increased infiltration, vascular permeability, and inflammatory cytokines and chemokines [14]. Our unilateral IR mice demonstrated that gene expression of renal NGAL and IL-6 in the contralateral kidney was upregulated in spite of no change in renal function. Thus, IRI-induced inflammatory cytokines and chemokines from the resected kidney (IRI kidney) cause tubular damage in the contralateral kidney as an organ crosstalk. Further, only acute elevation of contralateral urinary β_2_MG after clamping renal artery, but not the resected kidney-related injury markers and RAPN-related clinical parameters including blood loss, was correlated to chronic changes in renal function at 1 month after RAPN. Our finding indicates that renal prognosis after RAPN might depend on contralateral kidney’s tubular damage. Focusing on contralateral kidneys should be required to prevent chronic kidney injury after RAPN.

Regarding surgical treatment for small-diameter renal cancer, the only RCT by EORTC-30904 in 2011 showed that partial nephrectomy did not significantly differ from radical nephrectomy in overall survival in terms of cancer control [1]. Also, 26% of patients with small-diameter renal cancer are reported to have CKD even if their preoperative serum creatinine levels are within normal range; thus, there have been concerns regarding nephrectomy-induce additional renal damage [15]. However, partial nephrectomy has been shown to reduce the risk of CKD [16], all-cause and non-cancer related mortality[17], the prevalence of cardiovascular events [18], and other factors associated with mortality when compared to nephrectomy[3]. In addition, RAPN is a surgical assist device that can compensate for the weakness of manual manipulation in LPN, including tumor removal and parenchymal suture, and is comparable to LPN in terms of radical cure, shorter WIT, and lower complication rate [19]. The perioperative results of our study showed no conversion to laparotomy or grade 3 or higher complications, all of which were comparable to the previous report [20]. As demand of RAPN will continue to rise, further investigation will be necessary to create the precautions and therapeutic interventions to halt RAPN-related chronic kidney injury.

There are several limitations to this study. First, the number of patients is small, and further study is needed to accumulate more cases. Second, the mechanism of the effect of IRI on the contralateral kidney and the long-term effect of RAPN on renal function are unknown. Thirdly, it is generally difficult to collect urine from the healthy kidney in standard RAPN. However, since no increase in β_2_MG was observed in the urine collected from the resected kidney, measuring β_2_MG in bladder urine may reflect the urinary β_2_MG levels of the contralateral kidney. Fourthly, to acquire adequate statistical power (1-β >0.8), our clinical study was supposed to include 34 patients by using a sample size calculation tool [21]. However, it was difficult to collect the number of patients who received RAPN for RCC in a single center, the point of which is one of our limitations. Finally, this study did not include healthy individuals as controls, which may introduce potential bias. Therefore, large-scale, longitudinal, and prospective placebo-controlled studies should be conducted in the future.

## Conclusions

IRI after renal artery clamping during RAPN occurs in the resected kidney, which, in turn, extend the kidney damage to the contralateral kidney. Urinary β_2_MG excreted from the contralateral kidney after clamping renal artery may become a potent biomarker to predict the new onset of chronic kidney injury after RAPN.

## Supporting information

S1 File CONSORT Flow diagram. (DOCX)

S2 File This is the file including all data underlying the findings described in this manuscript. (XLSX)
