# Supplementary material for: The impact of ischemic reperfusion injury on contralateral kidneys and the determinants of renal prognosis after robot-assisted partial nephrectomy
Source: PLoS One. 2025 Apr 15;20(4):e0321769. doi: 10.1371/journal.pone.0321769 (PMC11999104; doi:10.1371/journal.pone.0321769)
Supplement: S1 File — (DOCX) [file pone.0321769.s001.docx]

**S Fig 1** CONSORT Flow diagram

Excluded (n= 0)

## Analysis

Follow up (n=23)

Excluded (n= 0)

Excluded (n= 0)

## Follow-Up

## Allocation

Allocated to surgery (n= 23)

## Enrollment

Excluded (n= 0)

After surgery (n=23)

Before surgery (n=23)

Assessed for eligibility (n= 23)
